# Suboptimal Adherence to Hereditary Cancer Risk Management Guidelines: A Cohort Study of High-Risk Individuals in Newfoundland and Labrador, Canada

**DOI:** 10.3390/curroncol33040184

**Published:** 2026-03-26

**Authors:** Holly Etchegary, Rebecca Puddester, Zhiwei Gao, Vanessa Francis, Mike Warren, T. Nadine Burry, Melanie Seal, Michael Woods, Kathy Watkins, April Pike, Susan Avery, Jerry McGrath, Andree MacMillan, Lesa Dawson

**Affiliations:** 1 Population Health and Applied Health Sciences (Clinical Epidemiology), Faculty of Medicine, Memorial University of Newfoundland, St. John’s, NL A1B 3V6, Canada; zgao@mun.ca; 2 Faculty of Nursing, Memorial University of Newfoundland, St. John’s, NL A1B 3V6, Canada; rjp823@mun.ca (R.P.);; 3 Patient Partner, St. John’s, NL A1B 3V6, Canada; 4 Department of Chemistry, Memorial University of Newfoundland, St. John’s, NL A1C 5S7, Canada; 5 Discipline of Oncology, Faculty of Medicine, Memorial University of Newfoundland, St. John’s, NL A1B 3V6, Canada; 6 Cancer Care Program, Newfoundland and Labrador Health Services, St. John’s, NL A1B 3V6, Canada; 7 Division of Biomedical Sciences, Discipline of Oncology, Faculty of Medicine, Memorial University of Newfoundland, St. John’s, NL A1B 3V6, Canada; 8 Centre for Nursing Studies, Newfoundland and Labrador Health Services, St. John’s, NL A1A 1E5, Canada; 9 Family Medicine, Faculty of Medicine, Memorial University of Newfoundland, St. John’s, NL A1B 3V6, Canada; 10 Gastroenterology, Faculty of Medicine, Memorial University of Newfoundland, St. John’s, NL A1B 3V6, Canada; 11 Provincial Medical Genetics Program, Newfoundland and Labrador Health Services, St. John’s, NL A1B 3V6, Canada; 12 Gynecologic Cancer Prevention and Survivorship Clinic, Division of Gynecologic Oncology, Vancouver General Hospital, Vancouver, BC V5Z 1M9, Canada; lesa.dawson@vch.ca; 13 Department of Obstetrics and Gynecology, Faculty of Medicine, University of British Columbia, Vancouver, BC V6T 1Z2, Canada

**Keywords:** hereditary cancer syndrome, *BRCA*, Lynch syndrome, risk management, screening, cancer, compliance

## Abstract

Hereditary cancer syndromes (HCSs), including *BRCA 1/2*-associated hereditary breast and ovarian cancer (HBOC) and Lynch syndrome (LS), confer substantial lifetime cancer risks, yet adherence to recommended risk-management strategies remains variable. This population-based retrospective cohort study examined cancer prevention practices, outcomes, and predictors of cancer occurrence among 476 *BRCA* and LS carriers identified through the Provincial Medical Genetics Program in Newfoundland and Labrador, Canada (2001–2022). Linked genetic, surgical, screening, and cancer registry data were evaluated, with adherence assessed during two intervals (2018–2020 and 2020–2022) based on NCCN guidelines. Participants were predominantly female (69%), with a mean age of 48.5 years at genetic testing; nearly 70% of primary cancers were already diagnosed at the time of testing. *BRCA* carriers demonstrated higher uptake of breast MRI (58–61%) and risk-reducing salpingo-oophorectomy (63–66%) compared with LS carriers’ colonoscopy uptake (42–44%). In univariate analyses, non-adherence during 2018–2020 was associated with increased odds of cancer after testing (OR ≈ 4.43, *p* < 0.001); this remained significant in the multivariate model (OR = 8.70; *p* = 0.0004). Individuals who did not follow recommended risk-management guidelines had nearly nine times greater odds of developing cancer after genetic testing than those who fully adhered to guidelines. Older age at referral (OR = 1.07/year, *p* < 0.001) also increased the odds of developing cancer. Study findings indicate that late referral and pre-existing cancers diminish the preventive impact of guideline-based risk management. System-level initiatives to promote earlier genetic testing, enhance cascade outreach, and strengthen surveillance pathways are needed to optimize cancer prevention and earlier cancer detection in high-risk populations.

## Simple Summary

This study looked at how well people with inherited cancer risk conditions—*BRCA*-related hereditary breast and ovarian cancer and Lynch syndrome—follow recommended cancer screening and prevention over time in Newfoundland and Labrador. Researchers followed 476 people identified through the Provincial Medical Genetics Program and linked their genetic test results with health records between 2001 and 2022. Overall, many people were already diagnosed with cancer by the time they received genetic testing, which limited the chance that screening could prevent cancer or allow earlier cancer detection. Around half of those with *BRCA* followed all recommended prevention steps, such as breast MRI or risk-reducing surgery. In contrast, fewer than half of those with Lynch syndrome had regular colonoscopy screening as recommended. The study found that people who followed cancer prevention guidelines had a lower chance of developing cancer than those who did not follow guidelines. Older age at referral for genetic testing was also a strong predictor of cancer. These findings highlight the need for earlier genetic testing, better follow-up, and more support to help high-risk individuals and families access preventive care before cancer develops.

## 1. Introduction

Hereditary cancers comprise up to 10% of the global cancer burden, with higher prevalence in certain cancer types [1,2,3]. Hereditary cancer syndromes (HCSs) confer a very high risk of malignancies in multiple organ systems, often at young ages [4]. Guidelines for the management of HCSs, such as hereditary breast ovarian cancer syndrome (HBOC; *BRCA 1* and *BRCA 2* genes) [5,6,7] and Lynch syndrome (LS; *MLH1*, *MSH2*, *MSH6*, and *PMS2* genes) are available [8,9,10,11] and regularly updated in response to genomic discoveries (e.g., moderate penetrance variants) and risk-management evidence, e.g., [5,8].

Guidelines vary by pathogenic variant (PV); however, high-risk individuals are generally recommended to have early and increased cancer screening. Cancer-type specific surveillance is suggested, including a range of comprehensive clinical exams and imaging such as MRI, colonoscopies, and mammograms. Guidelines can include pharmacological recommendations and reproductive decision-making discussions. Guidelines also recommend considering risk-reducing prophylactic surgery for associated cancers where evidence-based screening is limited (e.g., removal of ovaries after completion of childbearing in carriers of *BRCA 1/2* PVs) [5].

Adherence to cancer risk management can improve morbidity and mortality in PV carriers [12,13,14,15,16,17], with growing evidence for the efficacy of cancer genomics in treatment decisions [18,19]. Identification of people with HCSs also enables cascade screening of their at-risk relatives. Globally, however, there is unequal access to genetic testing, with increased demands on clinical genetics services [20,21,22]. This contributes to a lack of follow-up of patients with a HCS to ensure they are receiving life-saving risk management and an assessment of their long-term clinical outcomes [23,24,25,26,27,28].

Available evidence suggests risk management is suboptimal, even within the well-studied populations of HBOC and LS. For example, among 227 LS variant carriers, 68% of colonoscopies were adherent to recommended screening intervals [29]; in a more recent survey study of 197 LS-affected individuals, 28% reported that they were not adherent to screening intervals, while another 10% reported they had considered quitting or delaying surveillance [30]. Even in studies reporting generally high adherence to colonoscopy, adherence to extracolonic screening recommendations, such as endoscopy, was less optimal [27,31], and much lower for subsequent colonoscopy screening after the initial screen [27]. In our study site, adherence to recommended colonoscopic screening in LS carriers has not been described since 2012 [32]. At that time, a little over 40% of LS carriers were adherent to having a colonoscopy within 1–2 years of their prior screen.

In HBOC populations, adherence to risk management is also variable. Globally, reviews report ranges for the uptake of risk-reducing salpingo-oophorectomy (RRSO) of 17–98.5% [33] and ~36–71% [34]. Uptake is influenced by a host of sociocultural, provider, patient, and health system factors, which might explain the variability [33,35,36]. We found an RRSO uptake rate of ~75% in *BRCA* carriers identified up to 2017 [37], similar to rates reported elsewhere with dedicated clinic services and specialist counseling [38]. There was an uptake of ~61% for breast MRI and mammogram recommendations [37], with adherence to breast MRI within an 18-month period being only ~40%.

A survey study of *BRCA* carriers reported that 49% were adherent to mammograms and clinical breast exams, while just over half (51%) had risk-reducing mastectomy (RRM) [39]. Our reported RRM rate of 39.0% is similar to other reported Canadian rates (38.0–41.2%) [34,40]. Wide variation in the proportion of *BRCA* carriers choosing prophylactic surgery over screening has been reported, ranging from 13–53% for RRSO and 0–54% for RRM. In that review, adherence to annual mammography recommendations ranged from 57–93% [41].

Individuals with HCSs face high, lifelong cancer risks. Yet few studies have followed these patients over time. As a result, there is limited understanding of their adherence to risk-management recommendations or their cancer outcomes. In Newfoundland and Labrador (NL), Canada, genetic counseling and testing for high-risk individuals are provided through the Provincial Medical Genetics Program (PMGP). After a positive genetic test, carriers receive a letter outlining risk-management recommendations to share with their primary care providers and family. The PMGP refers carriers to specialists as needed (e.g., medical and gynecologic oncology, urology), but there is no system to track whether they attend these appointments or complete recommended surveillance. Ongoing care and risk management are left to the patients themselves, their family physicians, and a small number of specialists with expertise in hereditary cancers in the province.

In this retrospective cohort study, we aimed to (1) describe the nature and extent of cancer surveillance, cancer prevention interventions, and cancer outcomes in *BRCA* and LS carriers in the Eastern Canadian province of Newfoundland and Labrador (NL) and (2) document the association of risk-management adherence with cancer outcomes.

## 2. Materials and Methods

### 2.1. Study Population

**Figure 1:**
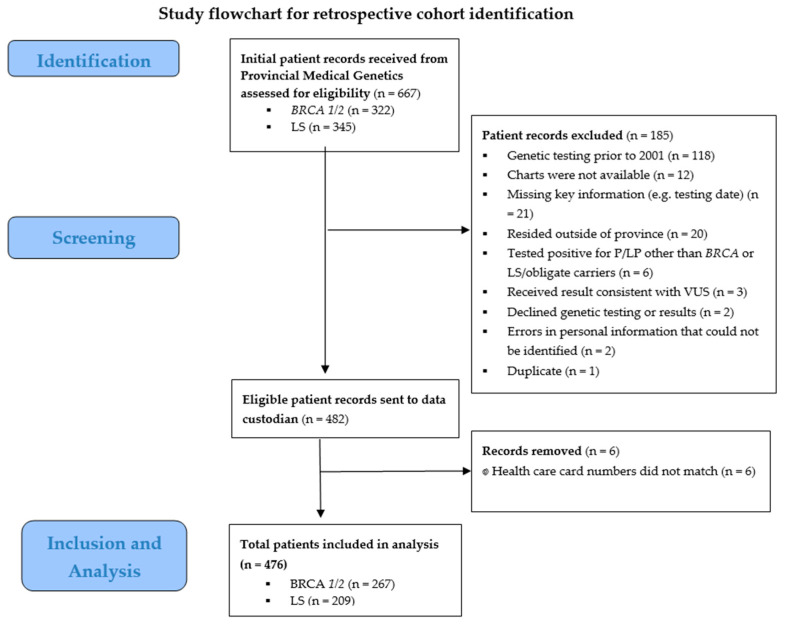
Study flowchart for retrospective cohort identification.

A research assistant working with a genetic counselor at the Provincial Medical Genetics Program (PMGP) compiled a spreadsheet of all *BRCA* and LS carriers, living and dead, from electronic and paper medical records for the study period (January 2001–March 2022, the latest data available during the study). Exclusion criteria were applied (see Figure 1).

### 2.2. Study Variables Extracted

Variables extracted from the PMGP’s patient database included: provincial health card identification number (called MCP# in NL), sex, date of genetic testing and date of results disclosure, variant identified, referring physician, and whether or not the carrier was a proband (Yes/No, where proband refers to the first person to receive genetic testing in a family).

Digital Health NL is the primary provincial data custodian that collects and maintains a wide range of health data. These include provincial surgical and screening data, the chronic disease registry, imaging and lab data (e.g., MRIs, PSA screening), prescription data, as well as patient demographic information. A second data custodian is Newfoundland and Labrador (NL) Health Services, the custodian for the provincial Cancer Care Registry, which maintains cancer incidence, tumor, and staging data.

Variables requested and extracted by Digital Health NL for this study included: patient diagnoses of diabetes, hypertension, ischemic heart disease, and myocardial infarction (to allow an estimate of number of comorbidities); date and type of hereditary cancer syndrome (HCS) prophylactic surgeries (RRSO, RRM, hysterectomy, colectomy and gastrectomy); date and type of HCS screening (colonoscopy, prostate antigen screening); and date and type of HCS imaging (breast MRI, mammogram).

The Cancer Care Registry (CCR) extracted the date and type of all primary cancer diagnoses and cancer stage, advising that staging data were incomplete and not of good quality. Blood cancers are not part of the CCR, and those data were not available. Cancer descriptions were derived from typology codes of the International Statistical Classification of Diseases for Oncology, third edition (ICD-O-3) [42].

### 2.3. Data Linkage and Final Study Dataset

Digital Health NL used patients’ MCP# to link all data. After linkage, each patient was given a unique study ID#, and the identifying health card number was removed. De-identified, linked data were provided to the study team in multiple SPSS files. Team members (NB, HE) carefully reviewed all files and created a single SPSS file for analysis. The acquisition of study data from all custodians took approximately one year (Fall 2022–Fall 2023), with another 6–8 months to clean, screen, and verify the study variables with data custodians before finalizing the dataset for analysis.

### 2.4. Constructing the Adherence Variable

Adherence to risk-management guidelines was defined over two time periods to account for the timing of the COVID-19 pandemic. Eligibility for each risk-reducing intervention was assessed from 1 March 2018 to 31 March 2020 and 1 April 2020 to 31 March 2022 according to NCCN guidelines, accounting for any changes in recommendations over the study period (e.g., PSA screening for males with a *BRCA 2* mutation was recommended to begin at age 45 in earlier guidelines, but in 2021, the recommended starting age was 40) [5,8].

### 2.5. BRCA Carriers

Females between the ages of 25 and 75 and 30 and 75 were considered eligible for breast MRI and mammograms, respectively, if they had breasts. Those with breasts removed either prophylactically or because of a cancer diagnosis were excluded. Breast MRI screening was considered adherent if undertaken within 18 months of our study periods. While guidelines suggest annual screening, we allowed additional time to account for known difficulties of booking MRIs in our province [23,24,37]. Carriers eligible for RRM were females 25 to 75 with breasts at the time of genetic testing and at any time up to and including our adherence periods. Eligibility for RRSO differed slightly depending on the specific *BRCA* pathogenic variant. Females between 35 and 75 years with a *BRCA 1* variant and between 40 and 75 years with a *BRCA 2* variant, with ovaries at the time of genetic testing, were considered eligible for RRSO at any time from genetic testing to the time of our adherence periods.

Male carriers were considered eligible for PSA screening if they carried a *BRCA* *1* or *2* variant and were 45 years of age or older during our first adherence period, or 40 years of age or older during our second.

*BRCA* carriers were categorized into three levels of adherence—Not adherent, somewhat adherent, and fully adherent—while LS carriers were categorized into fully adherent or not adherent. Table 1 provides key criteria for the adherence categorizations.

### 2.6. Documenting Adherence Decisions

Each carrier’s unique surgical, screening, and imaging history was carefully reviewed over both adherence periods; colonoscopies were further reviewed in LS carriers over all the years of the study data. Careful review was required to account for exclusions based on genetic testing date, death date, and age to ensure eligibility criteria were met for all screening and prevention modalities. For example, there were cases where a patient was below the recommended age to start screening during the first adherence period, but was of age during the second. This person would have been excluded from the first period but included in the second. Similarly, there were cases where a person had genetic testing too close to the first period to allow time for screening, but would have been included in the second, or cases where a person died during the second measurement period but would have been alive during the first and was included in the first adherence period. For these reasons, adherence numbers were slightly different between the two periods. These decisions, however, allowed usage of the maximum amount of data and were reflective of the complex reality of defining adherence from secondary data. A detailed file of inclusion and exclusion decisions for each carrier was retained by HE; this allowed repeated checking of numbers during data cleaning, coding, and analysis to ensure accurate inclusion/exclusion of carriers in adherence measurement periods.

### 2.7. Data Analysis

Descriptive statistics were used to describe the study population, adherence to risk management, and cancer outcomes. Differences in population characteristics between *BRCA* and LS carriers were examined by independent *t*-tests, ANOVAs, or correlations for continuous variables, and chi-square tests or Fisher’s Exact (where appropriate) for categorical variables (IBM SPSS Statistics for Windows, Version 29.0. Armonk, NY, USA). To identify significant predictors of cancer outcome, we used univariate and multivariate logistic regressions with generalizing estimating equations, which allow adjustment for clustering of individuals from the same family (SAS 9.4^®^ Statistical Analysis System Institute, Cary, NC, USA).

## 3. Results

### 3.1. Identifying Known Carriers

A total of 667 patient records were reviewed from the Provincial Medical Genetics Program to identify the study population; 185 were excluded, leaving 482 eligible records from 163 unique pedigree numbers. When these 482 patient records were sent to the data custodian, six had health card numbers that did not match and were removed, leaving 476 total patients in the study population (Figure 1).

### 3.2. Describing the Study Population

Table 2 describes the sample. Over half the study population carried a *BRCA* pathogenic variant (PV), and of these, 70% had a *BRCA 2* PV. Almost two-thirds of LS carriers had an *MSH2* variant. Almost 70% were female, with more female *BRCA* carriers than LS (Table 2). Most of the study population lived in towns with 5000 residents or more, although over 40% lived in towns with fewer than 5000 residents, a marker of rurality (Table 2). Of the 476 identified carriers, 91 were probands, with the remaining 385 being at-risk relatives. More *BRCA* carriers (*n* = 68) were probands than LS carriers (*n* = 23) (*p* < 0.001). Most pathogenic carriers had been referred to the Provincial Medical Genetics Program by their primary care physician, followed by through research studies and specialists (Table 2). Removing the small number of self-referrals, other, or unknown referral sources, revealed that almost half of *BRCA* carriers had been referred by their family doctors, with more LS carriers being referred through research studies (*p* = 0.022).

There were 393 patients living in 2022 (latest available study data), with more *BRCA* PV carriers (*n* = 55, 20.6%) having died compared with LS carriers (*n* = 28, 13.3%) (*p* < 0.05). Of living patients (*n* = 393), the mean age in 2022 was ~55 years old (SD = 14.4; range 22–90 years). For these patients, an average of 8.6 years had passed since genetic testing (SD = 5.1, range 0–21 years), with no difference among *BRCA* (8.2 years) and LS (9 years) carriers (*p* = 0.14).

At the time of genetic testing, the average age of the study population (*n* = 461) was 48.5 (SD = 15.3, range 17–87), with *BRCA* carriers older (50.2 years) than LS carriers (46.1 years) (*p* ≤ 0.005). Over half the sample had no comorbidities (54%), while roughly 30% of all *BRCA* and LS carriers had at least one comorbidity. There was no difference in the mean number of comorbidities between living *BRCA* (0.68) and LS carriers (0.57) (*p* = 0.21).

### 3.3. Cancer Outcomes in the Study Population

There were 203 primary cancers among the total study population (*n* = 476), with over half never having had cancer (57.4%); almost 30% of patients had one primary cancer (*n* = 138, 29%) (Table 2). The mean number of primary cancers was slightly higher in LS carriers (1.6) compared to *BRCA* carriers (1.3) (*p* = 0.009). Of the 203 primary cancers, the genetic testing date was missing for eight patients, leaving 195 for analysis. Almost 70% of these primary cancers were already diagnosed at the time of genetic testing (Table 2), with significantly more primary cancers diagnosed in *BRCA* carriers (*n* = 83) than in LS carriers at the time of testing (*n* = 51) (*p* < 0.001).

There was a wide variety of primary cancers, with breast and ovarian the most frequent in *BRCA* carriers and colon the most frequent in LS carriers. Of the 104 primary cancers in *BRCA* carriers, ~54% were breast cancers, while ~13% were ovarian. Just over one-third (34%) of the primary cancers in LS carriers were colon cancer. Including all 203 cancers in the study population, the mean age at diagnosis was around 52 years, with no difference in age at diagnosis among LS and *BRCA* carriers (Table 2).

### 3.4. Cancer Stage

Including all 203 primary cancers in the study population, staging data were missing for 55 (27%). Of the remaining 148 cancers, 12 were Stage 0 (8.1), 50 were Stage 1 (33.8%), 49 were Stage 2 (33.1%), 25 were Stage 3 (16.9%), while 12 were Stage 4 (8.1%). While *BRCA* carriers had higher numbers of Stage 3 and 4 cancers (*n* = 27) than LS carriers (*n* = 10), this was not significantly different (*p* = 0.08). Of the 61 cancers diagnosed after genetic testing with tumor staging data available (*n* = 44), most were Stage 0, 1, or 2 (*n* = 6, 18, 13, respectively), while only seven were Stage 3 or 4.

### 3.5. Adherence to Risk Management

#### 3.5.1. BRCA Mutation Carriers

Table 3 displays the counts of living *BRCA* participants eligible for, and undergoing, surgical and screening interventions during the two adherence periods.

Approximately 70% of eligible females had at least one mammogram during the two study periods, while around 60% had a breast MRI; 35% of eligible females had RRM, while roughly two-thirds had RRSO by the time of the adherence periods. Of the males, nearly or over two-thirds had at least one prostate antigen screen (PSA) during the study periods.

Using this information to categorize the levels of adherence of *BRCA* carriers revealed that nearly or just over half were fully adherent to risk-management guidelines, while over a third were at least somewhat adherent. Over 10% were not adherent at all (Table 3).

#### 3.5.2. Adherence—LS Carriers

There were 209 patients in the LS cohort. Colonoscopy uptake was first examined with a simple count of the total number of colonoscopies received after genetic testing for each person with LS, including those who had died. Patients whose death date was too close to genetic testing to allow time to receive a colonoscopy, whose genetic testing date was unknown, or who had genetic testing in 2020 or after, were excluded (as this would not have allowed enough time to receive a colonoscopy before our study end date). This resulted in 37 exclusions, leaving 172 carriers. The mean number of colonoscopies received by these carriers since their genetic testing was 2.43 (SD = 2.41), with a median of 2 (Range 0–12).

After applying age, genetic testing date, and death exclusions, 155 and 154 living patients were eligible for colonoscopies during the two study periods, respectively. Just over 40% were fully adherent, receiving a colonoscopy during each adherence period (Table 4).

Removing the 62 “somewhat adherent” *BRCA* carriers revealed a clear significant difference between patients with *BRCA* and LS who were fully adherent to risk-management practices. In the first compliance period, 80% of *BRCA* carriers were fully adherent vs. 44% of LS carriers (*p* < 0.001), which was almost identical to the fully adherent percent (79% *BRCA*, 42% LS) in the second period (*p* < 0.001).

#### 3.5.3. Relationship Between Adherence Levels and Cancer Outcomes

Table 5 displays the results of the univariate logistic regression analyses of being diagnosed with cancer after genetic testing.

Univariate findings indicate that non-adherence to risk-management guidelines during 2018–2020 was significantly associated with higher cancer occurrence post-genetic testing (OR ≈ 4.43, 95% CI: 2.13–9.06, *p* < 0.001), whereas adherence during the second period was not statistically significant. Age at referral emerged as a predictor, with each additional year increasing the odds of cancer after genetic testing by ~4% (OR = 1.04, 95% CI: 1.02–1.06, *p* < 0.001). Referral source was also significant: patients referred by general practitioners had lower odds of cancer after genetic testing compared to those referred by specialists or through research studies (OR ≈ 0.16, 95% CI: 0.08–0.34, *p* < 0.001). *BRCA* carriers had higher post-genetic testing cancer odds than Lynch carriers (OR ≈ 0.35, 95% CI: 0.18–0.67, *p* = 0.0018). Number of comorbidities, sex, rurality, and proband status were not significant predictors of cancer diagnosis post-genetic testing.

Table 6 presents the results of the multivariate logistic regression model of cancer occurrence after genetic testing, after adjusting for demographic and clinical variables. Adherence during 2018–2020 was associated with marked differences in the odds of having a cancer diagnosis after genetic testing. Specifically, non-adherence to guidelines was associated with higher odds of post-testing cancer (OR = 8.70; 95% CI: 2.65–28.61; *p* = 0.0004), compared to full adherence.

By contrast and unexpectedly, the pattern reversed in the second adherence period: non-adherent carriers had lower odds of developing cancer after genetic testing than fully adherent carriers (OR = 0.30; 95% CI: 0.10–0.94; *p* = 0.038). However, upon examining the overall statistical test for this variable (Type 3 *p*-value), it was not statistically significant (*p* = 0.0743). This type of *p*-value is used when a variable has more than two groups, and tests whether there is an overall difference across all groups combined.

Age at referral, however, remained a consistent predictor: each additional year of age increased the odds of developing cancer post-genetic testing by ~7% (OR = 1.07; 95% CI: 1.03–1.10; *p* < 0.001). In terms of referral source, family doctor referral was associated with lower odds of post-genetic testing cancer (OR = 0.17; 95% CI: 0.05–0.52; *p* = 0.002) compared to referral through a research study or specialists. Other factors, including sex, rurality, proband status, *BRCA* vs. LS status, and number of comorbidities, were not significant in the multivariate model.

## 4. Discussion

The aim of this study was to describe trends in risk-management practices among *BRCA* and LS PV carriers and identify gaps in uptake to inform strategies for cancer risk reduction. We also describe cancer outcomes in this cohort and their relationship to risk management.

A notable finding was that ~70% of the primary cancers in this cohort were already diagnosed before, or on the same date, as genetic testing, suggesting that many individuals were referred for testing because they had a cancer suspected to be genetic. Thus, many were beyond the window where screening could help identify cancer early or where risk-reducing surgeries could prevent cancer. This finding underscores the importance of early genetic testing and timely referral, as late identification limits the potential impact of adhering to risk management on cancer prevention.

Our findings are similar to a recent Canadian study of *BRCA* carriers that reported “major gaps” in identifying these individuals before the development of cancer [43]. In that cohort, women with cancer accounted for the greatest proportion of people accessing genetic testing over a 10-year period, and the age at genetic testing was 54 and increasing, well beyond the age at which breast cancers typically develop in *BRCA* carriers [44]. While the mean age of our *BRCA* cohort at the time of genetic testing was lower (~50 years), it was still beyond the age at which cancers develop in many PV carriers.

Cascade testing of at-risk relatives provides an opportunity to identify high-risk individuals before cancer develops, yet uptake remains suboptimal, e.g., [45,46]. Some authors have called for population-based genetic testing for hereditary cancer syndromes, partly because of the low uptake of cascade testing and the inefficiencies in identifying high-risk individuals; others note that current referral criteria for genetic testing are too focused on family history, and known to miss many high-risk individuals [25,43,47,48].

In our cohort, most individuals were at-risk relatives (with only ~18% being probands); however, the uptake of risk management was suboptimal in this group and in some cases, quite low. For example, in LS carriers, the median number of colonoscopies received was only two, despite nine years having passed since genetic testing, and they were significantly less likely to be fully adherent than *BRCA* carriers. This said, ~40% of *BRCA* carriers were not having breast MRIs as recommended by guidelines, and ~30% were not having mammograms. We recognize that screening adherence can be particularly difficult for pre-menopausal women (e.g., screening is often held during pregnancy and breastfeeding, and breast MRIs are timed with monthly cycles, making re-booking difficult in a resource-constrained system). Nonetheless, these findings suggest many missed opportunities for cancer prevention or earlier cancer detection and the need for interventional research to create and test interventions to increase the uptake of risk-management modalities.

There is already a large literature on barriers to colonoscopic screening specifically, [49,50,51] and barriers to risk management for hereditary cancers generally, e.g., [24,52]. There are comparatively fewer studies that create and test interventions to address known barriers. If we are to fully utilize the power of genetic testing to reduce cancer burden, health system and research efforts should focus on developing programs and policies that better identify high-risk individuals before cancer develops. We recommend these be co-developed with patients, families, and providers in order to prioritize known barriers in light of care pathways, and logistical and budget restraints in local contexts. In our jurisdiction, recent health policy strategic direction [53] recommends the creation of specialty cancer preventative services for high-risk families, both to accelerate access to the molecular testing required for standard-of-care targeted therapies [54] and facilitate coordinated follow-up of high-risk patients. Such efforts may improve cancer prevention in high-risk populations, although this would need ongoing evaluation.

Multivariate analyses revealed that older age at referral was consistently associated with higher odds of post-genetic testing cancer, underscoring that later identification of hereditary cancer syndrome (HCS) carriers likely diminishes the potential impact of preventive surveillance and risk-reducing interventions. This aligns with our broader cohort observation that many cancers were already present at the time of testing. Together, these findings reinforce the need to accelerate upstream case-finding and cascade testing so that high-risk individuals are identified earlier in the disease course. Improving awareness and education within primary care settings could help with earlier identification and testing of high-risk individuals. This is supported by the finding that unaffected carriers referred to genetic testing by general practitioners had lower odds of developing cancer post-testing compared to those referred by specialists or research studies. By the time a patient is referred for genetic testing by a specialist provider, typically oncology, they may already have cancer.

Unexpectedly, adherence effects differed by period. During 2018–2020, non-adherence to risk-management guidelines was associated with substantially higher adjusted odds of post-testing cancer relative to full adherence, consistent with the protective intent of guideline-concordant management and in line with prior research, e.g., [13,14,15,16]. In contrast, during 2020–2022, the model estimates suggested lower odds of cancer diagnosis among non-adherent carriers versus fully adherent carriers; although this was not significant when observing the overall statistical test for this variable. This counterintuitive pattern might reflect a combination of period-specific biases and structural disruptions. For example, COVID-19-related service interruptions may have altered both surveillance opportunities and the timing of cancer detection. Selection bias is also plausible: individuals developing cancer may be triaged into oncology pathways (and discontinue routine surveillance counted as “adherence”), while those without events could appear “non-adherent” within the narrow observation window. Finally, because both adherence periods were highly correlated and entered the regression model simultaneously, this could have affected the model estimates for the second adherence period.

The multivariate model also revealed that the referral pathway was a significant predictor of cancer outcomes. Compared with research-referred carriers, those referred by general practitioners had lower adjusted odds of post-testing cancer, while specialist referral did not differ significantly from research referral. One interpretation is that primary care identification may occur earlier in the risk trajectory, whereas specialist referrals are more likely for individuals already undergoing diagnostic work-up, thereby compressing the opportunity for prevention. Although non-significant, the directionality of the specialist estimate is compatible with this clinical reality.

Collectively, the adjusted multivariate model suggests that cancer outcomes are shaped by when and how carriers enter the genetic care pathway and not just by cross-sectional adherence snapshots alone. Clearly, adhering to risk-management guidelines has a positive impact on cancer outcomes, as demonstrated by the large odds ratio in the multivariate model. However, system policies that enable the identification of carriers at younger ages, streamline primary care-based risk assessment, and strengthen cascade outreach are likely to yield gains in prevention in addition to those efforts focused solely on improving adherence within currently late-identified cohorts. Given the period-specific adherence signal and the modest precision of some estimates, confirmatory analyses in larger cohorts and prospective registry-based follow-up are indicated to clarify causal effects and mitigate measurement artifacts introduced by pandemic-era service disruptions.

The relationship between genetic testing and subsequent protective health behaviors, such as cancer screening, is complex and likely mediated by many factors, including individual risk perception, health literacy, access to health services, and geographic and financial constraints [52,55,56]. As in our data, recent studies [56] failed to find a simple relationship between genetic testing and protective cancer screening behaviors, such that those identified as high-risk would be most adherent to cancer risk-management behaviors and thus, have better cancer outcomes. Overall, our findings add to the growing understanding of the complex relationship between genetic testing for inherited cancer risk and subsequent screening behaviors, highlighting the need for ongoing research to better identify moderating and mediating variables, including the social and structural determinants of health.

### Limitations

While the data reflect a population cohort and significant efforts were made in coding to utilize all the available data, the sample size is small, impacting statistical power to detect relationships among study variables. Confirmatory analyses with larger cohorts are needed. This study was retrospective using secondary data. Data quality was not ideal, and in some cases, particularly poor (e.g., cancer staging data). Multiple data custodians, differences in data coding and definitions, and lengthy data acquisition periods all contributed to suboptimal data quality. There were also unavailable data, such as personalized screening recommendations (e.g., based on family history) and other screening data that should have been included in definitions of adherence (e.g., endoscopy screening, annual dermatology checks). Furthermore, for both *BRCA* and LS carriers with incurable disease, screening typically discontinues, impacting adherence numbers post-cancer diagnosis. Again, the secondary data did not allow assessment of individualized screening recommendations or behavior. We retained cancer-affected carriers in the cohort since they remain at elevated risk for subsequent primary cancers and continue to be eligible for guideline-based surveillance. However, their surveillance pathways can be influenced by oncology follow-up schedules, treatment-related considerations, or specialist-driven care plans, which could diverge from the guideline-based management recommendations given to unaffected carriers. Thus, differing clinical trajectories of affected and unaffected pathogenic variant carriers can influence observed adherence rates. Given our access was solely to a secondary dataset, not individual patient records, we do not know what patient-specific protocols may have been given to any individual carrier.

The secondary data also did not include self-reported gender or ethnicity. Ideally, future research that is prospective in nature and supported with a high-risk patient registry would allow better exploration of the relationship between risk-management adherence and cancer outcomes. As the use of multigene panel testing for the diagnosis of hereditary cancer syndromes increases, a broader spectrum of hereditary cancer risk variants continues to be identified (e.g., *PALB2, ATM, CHEK2, BRIP1*) [57,58,59,60]. These variants may be important in the identification of other carriers who could benefit from targeted therapies, surveillance, cancer prevention opportunities, and cascade testing [58,60]. While the current study focused on adherence to risk-management guidelines in the well-studied *BRCA* and LS populations, there is a need for ongoing research to inform appropriate surveillance strategy development and refinement for these less well-known variants. Prospective research that follows carriers of variants beyond *BRCA* and LS would be useful to inform clinical management and determine the effectiveness of risk-management guidelines as they evolve [28,60].

Finally, while these data are useful to describe risk management and cancer outcome trends over defined periods, qualitative research would be useful to help understand why adherence was suboptimal, thus providing targets for interventional research.

## 5. Conclusions and Recommendations

The study findings support the development of policies and practices that enable and support carriers’ adherence to risk-management guidelines. In one of our adherence periods, people who did not follow recommended risk-management guidelines were almost nine times more likely to be diagnosed with cancer after their genetic test than carriers who followed all recommended guidelines.

The findings also suggest that improving timely identification and referral of high-risk individuals should be a priority, as older age at referral was a predictor of cancer occurrence after genetic testing, while GP-initiated referrals were associated with lower post-genetic testing cancer odds. Implementing streamlined pathways for earlier genetic risk assessment in primary care, targeted provider education, enabling cascade testing of relatives, and reducing wait times for genetics and surveillance services are critical system considerations. The low colonoscopy adherence in LS patients underscores an urgent need for dedicated, navigation-supported LS surveillance clinics, reminder systems, or other barrier-reducing interventions. At the health system level, persistent gaps in the timely identification of high-risk patients and suboptimal uptake of risk-management interventions are important since genetic testing and screening in HCS are cost-effective [61,62,63]. Scaling direct-to-family cascade outreach and registry-based follow-up could yield meaningful prevention benefits, especially if coupled with equity-focused strategies that address geographic and access constraints.

## Figures and Tables

**Table 1:** Definitions of adherence to risk management in *BRCA* and LS carriers.

Carrier Type	Adherence Level	Definition/Categorization
*BRCA*—Female	Not adherent	Did not undergo RRSO or RRM and had no breast screening (mammogram or MRI) during study periods.
	Somewhat adherent	1. Had RRSO, but no breast screening of any kind; OR had any breast screening (mammogram or MRI) but no RRSO; OR had RRM, but not RRSO; OR 2. Had RRSO and mammograms, but not breast MRI, and had not had RRM.
	Fully adherent	Had RRSO and either regular breast MRI or had undergone RRM.
*BRCA*—Male	Not adherent	Had no PSA screening during adherence periods.
	Somewhat adherent	Had PSA screening at least once, but not annually as recommended.
	Fully adherent	Had annual PSA screening during adherence periods.
Lynch Syndrome	Fully adherent	Colonoscopy undertaken within each 2-year adherence period (March 2018–March 2020 and April 2020–March 2022).
	Not adherent	No colonoscopy within each 2-year adherence period.

**Table 2:** Demographic and clinical characteristics of *BRCA* and LS carriers in NL.

Characteristic	Overall	*BRCA 1/2*	LS	*p*-Value
**Total (*n*)**	476	56.1% (267)	43.9% (209)	
**Pathogenic variants**		BRCA 1: 30% (79) BRCA 2: 70% (188)	MSH2: 62% (130) PMS2: 29.6% (62) MSH6: 5.2% (11) MLH1: 2.8% (6)	
**Avenue of referral (*n* = 476)**				0.022
Family doctor	38.9% (185)	47.5% (118)	38.9% (67)	
Research study	25.8% (123)	21.4% (60)	36.6% (63)	
Specialist	25.3% (112)	28.2% (70)	24.4% (42)	
Self-referred	3.9% (19)	—	—	
Other	2.1% (10)	—	—	
Unknown	5.7% (27)	—	—	
**Sex**				0.001
Female	69.0% (329)	75.3% (201)	61.2% (128)	
**Alive in 2022**	393	212	181	0.04
**Age in 2022 (living patients)**				ns
Mean ± SD (years)	54.9 ± 14.4	55.6 ± 14.4	54.0 ± 14.4	
Range	22–90	22–88	23–90	
**Age at genetic testing (*n* = 461)**				<0.001
Mean ± SD (years)	48.5 ± 15.3	50.2 ± 14.8	46.1 ± 15.7	
Range	17–87	19–87	17–84	
**Number of primary cancers**	203	104 ($\bar{x}$ = 1.3)	99 ($\bar{x}$ = 1.6)	0.009
**Genetic testing at or after cancer diagnosis**	68.7% (134)	80.5% (83)	55.4% (51)	<0.001
**Number of comorbidities** *				ns
None	54.0% (257)	51.6% (138)	56.9% (119)	
1	29.0% (138)	29.9% (80)	27.7% (58)	
2	10.9% (52)	12.3% (33)	9.1% (19)	
3	4.6% (22)	4.4% (12)	4.7% (10)	
4	1.5% (7)	1.4% (4)	1.4% (3)	
**Health zone of residence (*n* = 474)**				<0.001
Eastern	67.5% (320)	76.7% (205)	55.5% (115)	
Central	12.5% (59)	12.4% (33)	12.6% (26)	
Western	17.7% (84)	8.7% (23)	29.5% (61)	
Labrador-Grenfell	2.3% (11)	2.2% (6)	2.4% (5)	
**Living in community ≤ 5000 residents (*n* = 474)**				ns
Yes	44.3% (201)	41.9% (112)	46.9% (98)	
No	55.7% (264)	58.1% (155)	55.1% (109)	
**Age at first cancer diagnosis (*n* = 203)**				ns
Mean ± SD (years)	51.8 ± 12.4	51.7 ± 11.6	51.9 ± 13.3	
Median	52	52	53	
Range	24–89	26–81	24–89	

* Includes diagnoses of diabetes, hypertension, ischemic heart disease, and myocardial infarction. ns = not significant

**Table 3:** Surgical and screening risk-management behaviors in *BRCA* carriers.

Screening and Surgical Management Practices	First Adherence Period March 2018–March 2020 # Adherent/# Eligible (%)	Second Adherence Period April 2020–March 2022 # Adherent/# Eligible (%)
Mammogram	60/84 (71%)	58/85 (68%)
Breast MRI	51/84 (61%)	49/85 (58%)
RRM	46/130 (35%)	46/131 (35%)
RRSO	72/115 (63%)	76/116 (66%)
PSA	22/36 (61%)	28/38 (74%)
Adherence level		
Not adherent	22 (12.7%)	23 (13.1%)
Somewhat adherent	62 (35.8%)	68 (38.6%)
Fully adherent	89 (51.4%)	85 (48.3%)
Total number of living participants	173 (*n* = 137 females)	176 (*n* = 138 females)

RRM Risk-Reducing Mastectomy; RRSO Risk-Reducing Salpingo-Oophorectomy; PSA Prostate Antigen Screening

**Table 4:** Adherence to colonoscopy recommendation in LS carriers.

Adherence to Colonoscopy Recommendations	First Compliance Period March 2018–March 2020 # Adherent/# Eligible (%)	Second Compliance Period April 2020–March 2022 # Adherent/# Eligible (%)
Adherence level		
Not adherent	87/155 (56.1%)	89/154 (57.8%)
Fully adherent	68/155 (43.9%)	65/154 (42.2%)
Total number of living participants	155	154

**Table 5:** Univariate logistic regression analyses.

Variable		OR (95%CI)	*p*-Value
Adherence period 2018–2020	0	4.43 (2.17, 9.06)	<0.001
	1	1.05 (0.35, 3.11)	0.9349
	2	1	
Adherence period 2020–2022			
	0	1.49 (0.78, 2.85)	0.2267
	1	0.47 (0.16, 1.38)	0.1709
	2	1	
Age at referral		1.04 (1.02, 1.06)	<0.001
*BRCA* vs. Lynch	*BRCA*	0.35 (0.18, 0.67)	
	LS	1	0.0018
Referral source	Specialist	0.78 (0.35, 1.73)	0.5415
	GP	0.16 (0.08, 0.34)	<0.001
	Research	1	
Rurality			
	Urban	1.46 (0.88, 2.43)	0.1412
	Rural	1	
Sex	Female	1.21 (0.74, 1.96)	0.4448
	Male	1	
Number of Comorbidities		1.20 (0.90, 1.58)	0.2141
Proband	Relative	0.95 (0.42, 2.15)	0.9008
	Proband	1	

Variable coding—Adherence: 0 = not adherent, 1 = somewhat adherent, 2 = fully adherent; *BRCA* vs. Lynch: *BRCA* = 1, Lynch = 2; Referral source: 1 = Specialist, 2 = Family doctor (GP), 3 = Research study; Rurality: 1 = living in a town with more than 5000 residents, 2 = living in a town with less than 5000 residents; Sex: 1 = female, 2 = male; Proband: 1 = at-risk relative, 2 = proband.

**Table 6:** Multivariate model of variables related to having a cancer diagnosis after genetic testing.

Variable		OR (95%CI)	*p*-Value
Adherence period 2018–2020	0	8.70 (2.65, 28.61)	0.0004
	1	1.86 (0.42, 8.15)	0.41
	2	1	
Adherence period 2020–2022			
	0	0.30 (0.10, 0.94)	0.0384
	1	0.36 (0.09, 1.41)	0.1425
	2	1	
Age at referral		1.07 (1.03, 1.10)	<0.001
*BRCA* vs. Lynch	*BRCA*	0.35 (0.10, 1.18)	0.0906
	LS	1	
Source of referral			
	Specialist	0.35 (0.09, 1.37)	0.1334
	GP	0.17 (0.05, 0.52)	0.0019
	Research	1	
Rurality			
	Urban	1.86 (0.83, 4.15)	0.1316
	Rural	1	
Sex	Female	1.16 (0.46, 2.97)	0.7521
	Male	1	
Proband	Relative	0.83 (0.14, 5.05)	0.8372
	Proband	1	
Number of Comorbidities		0.72 (0.42, 1.25)	0.2494

Variable coding—Adherence: 0 = not adherent, 1 = somewhat adherent, 2 = fully adherent; *BRCA* vs. Lynch: *BRCA* = 1, Lynch = 2; Referral source: 1 = Specialist, 2 = Family doctor (GP), 3 = Research study; Rurality: 1 = living in a town with more than 5000 residents, 2 = living in a town with less than 5000 residents; Sex: 1 = female, 2 = male; Proband: 1 = at-risk relative, 2 = proband.

## Data Availability

Due to the sensitive nature of clinical and genetic data and restrictions imposed by data custodians, the datasets generated and analyzed for the current study are not publicly available. Further data may be available from the corresponding author upon reasonable request.

## Abbreviations

The following abbreviations are used in this manuscript:

HCS	Hereditary Cancer Syndrome
HBOC	Hereditary Breast Ovarian Cancer
BRCA	Breast Cancer Gene (*BRCA 1* and *BRCA 2*)
LS	Lynch Syndrome
PV	Pathogenic Variant
P/LP	Pathogenic/Likely Pathogenic
VUS	Variant of Uncertain Significance
MRI	Magnetic Resonance Imaging
RRSO	Risk-Reducing Salpingo-Oophorectomy
RRM	Risk-Reducing Mastectomy
PSA	Prostate-Specific Antigen
ICD-O-3	International Classification of Diseases for Oncology, Third Edition
PMGP	Provincial Medical Genetics Program
CCR	Cancer Care Registry
NL	Newfoundland and Labrador
MCP#	Medical Care Plan identification number
NCCN	National Comprehensive Cancer Network
OR	Odds Ratio
CI	Confidence Interval
SD	Standard Deviation
GEE	Generalized Estimating Equation
